# Draft genomes of three aerobic methanotrophs from a temperate eutrophic fishpond

**DOI:** 10.1128/mra.00152-24

**Published:** 2024-03-25

**Authors:** Magdalena Wutkowska, Anne Daebeler

**Affiliations:** 1 Institute of Soil Biology and Biogeochemistry, Biology Centre CAS, České Budějovice, Czechia; DOE Joint Genome Institute, Berkeley, California, USA

**Keywords:** *Methylomicrobium*, *Methylobacter*, aquaculture, methane oxidation

## Abstract

Here we introduce draft genomes of three methanotrophs belonging to the *Methylococcaceae,* a family of typically fast-growing methane oxidizers. *Methylobacter* sp. Wu1, *Methylomicrobium* sp. Wu6, and *Methylobacter* sp. Wu8 were cultured from the top sediment collected from a shore of a eutrophic fishpond in the southern Czech Republic.

## ANNOUNCEMENT

Inland waters emit 398.1 ± 79.4 Tg CH_4_ year^−1^ (±95% CI) globally, with aquaculture systems being an increasingly important contributor to these emissions (1, 2). Methanotrophs, microorganisms that oxidize methane, play a pivotal role in mitigating these emissions by consuming up to 91% of the methane produced *in situ* before it can reach the atmosphere (3–6). Some of the fastest growing methanotrophs belong to the family *Methylococcaceae* (7). We cultured three new members of this family, designated as Wu1, Wu6, and Wu8, from sediments of the eutrophic fishpond Naděje located in the southern Czech Republic (49°02′12.9″ N, 14°26′04.4″ E).

### Culturing, sequencing, and data analyses

Fresh sediments collected from the fishpond on 25.02.2021 were incubated in 20–25 mL of nitrate mineral salt medium (NMS) (8) in 120 mL wide-neck bottles with butyl rubber stoppers and a headspace amended with 10%–15% of methane. The enrichments were incubated at room temperature and shaken at 90 rpm. After several transfers to fresh NMS, 50 µL of 10^−4^ and 10^−5^ dilutions of the culture was spread on 1% NMS agar plates. Material for Sanger sequencing was obtained from single colonies, which were then also transferred to fresh liquid NMS for cultivation. The colony PCR of the 16S rRNA gene with primers 27F (9) and 1492R(I) (10) was run over 30 cycles of 45 s of denaturation at 94°C, 30 s of annealing at 57°C, and 75 s of elongation at 72°C and was preceded by an initial step of 10 min at 94°C. The PCR products were cleaned with ExoSAP-IT (ThermoFisher Scientific) and sequenced (SeqMe, Czech Republic). The retrieved sequence with a quality score >20 (884–980 bp long) was used to assign taxonomy initially, by comparing them with MegaBLAST (11) against the RefSeq Representative Genomes database of NCBI.

As material for the genome sequencing, we used biomass pellets obtained by centrifugation at 4220 RCF for 10 min from 25 mL of three one-week-old optically dense cultures originating from single colonies. Total nucleic acids were extracted from these pellets according to the protocol of Angel et al. (12), which includes bead-beating with phenol. Extracted DNA was sequenced on an Illumina NovaSeq 6000 system (Joint Microbiome Facility, Vienna) with NEBNext Ultra II DNA Library Prep Kit for Illumina (New England Biolabs). Low-quality reads (SLIDINGWINDOW:4:20), short reads (<35 bp), and Illumina adapters were removed with trimmomatic 0.39 (13). Remaining reads were assembled with default settings using MEGAHIT 1.2.9 (14) and binned with default settings using metaBAT2 (15). Bins were assessed for taxonomy using single-copy gene analysis (SCG) in anvi’o 7.1 (16). In the three sequencing projects with Wu1, Wu6, and Wu8, we obtained three, two (1–200 Mb bin with no SCGs, not presenting characteristics of a plasmid), and one bin(s), respectively. The two additional bins in the Wu1 project (PRJNA1071020) were classified as *Pseudomonas* sp. and *Aquabacterium* sp. The bin qualities were assessed by CheckM v1.2.2 (17) and CheckM2 v1.0.1 (18). Assemblies were annotated with the NCBI prokaryotic annotation pipeline (19–21). The number of raw reads that mapped to each assembly was calculated using coverM with default settings (https://github.com/wwood/CoverM). Similarities to the closest related strains identified by both Sanger sequencing and SCG were further examined by comparing their genome-wide average nucleotide identities (ANI, here specifically OrthoANIu) with the three new genomes by using the online tool EZBioCloud (22).

### Genome characteristics and taxonomy

Basic description of the genomes’ characteristics, accession numbers, as well as the taxonomic identification are summarized in Table 1. The three genomes include a repertoire of methanotrophy-related genes typical for *Methylococcales* (Table 1), with two of them carrying more than one type of methane monooxygenase. These genomes are a valuable resource that widens the known genomic scope of metabolic versatility of gammaproteobacterial methanotrophs.

**TABLE 1:** Links for the deposited data and the major characteristics of the three genomes of new methanotrophs from a temperate eutrophic fishpond^*a*^

Genus	*Methylobacter* sp.	*Methylomicrobium* sp.	*Methylobacter* sp.
Strain	Wu1	Wu6	Wu8
BioProject	PRJNA1071020	PRJNA1051559	PRJNA1071006
SRA	SRR27843344	SRR27397163	SRR27843413
Total number of reads	71,768,776	113,477,650	49,392,754
NCBI RefSeq assembly	GCF_036553575.1	GCF_035916875.1	GCF_036440755.1
Genome coverage	415	2,200	1,085
coverM	25.41% (18,238,865 reads)	80.26% (91,076,379 reads)	91.26% (45,080,258 reads)
Size (bp)	4,395,384	4,147,043	4,156,109
Number of contigs	85	96	76
Contig N50	85.4 kB	67.7 kB	99 kB
Contig L50	19	19	14
Genes	4,052	3,923	3,796
Protein-coding sequences	3,969	3,788	3,721
GC content	52%	53%	50.5%
Completeness (M1/M2)	92.63%/100%	89%/100%	89.09%/98.44%
Contamination (M1/M2)	6.65%/0.36%	7.18%/0.19%	7.4%/0
16S rDNA taxonomy	98.9% id *M. marinus* A45 (NZ_KB912877.1) (23) 98.6% id *Methylobacter luteus* IMV-B-3098 (NZ_KE386569.1) (24)	97.6% id *M. lacus* LW14 (NR_042712.1) (25, 26)	99.55% id *M. tundripaludum* SV96 (NZ_JH109153.1) (27)
Genome taxonomy (SCG)	*M. luteus* (5/11)	*M. lacus* (11/21)	*M. tundripaludum* (16/21)
Genome taxonomy (ANI)	87% similarity to *M. luteus* IMV-B-3098 (GCF_000427625.1) (24)	86% similarity to *M. lacus* LW14 (GCF_000527095.1) (28)	85.2% *M*. *tundripaludum* SV96 (GCF_000190755.2) (27)
Genes/metabolic pathways	*pmoCAB*, *mxaFI*, H_4_MTP, H_4_F, RuMP, pSC	*pmoCAB*, *pxmABC, mxaFI*, H_4_MTP, H_4_F, RuMP, pSC	*pmoCAB*, *pxmABC, sMMO*, *mxaFI,* H_4_MTP, H_4_F, RuMP, pSC

^*a*^ CoverM values represent the percentage of raw reads that mapped to the assembled genome. Completeness and contamination values were obtained by checkM according to NCBI recommendation with a genus specific marker gene set (*Methylobacter* for Wu1 and Wu8, and *Methylomicrobium* for Wu6) (M1) and by checkM2 with default settings (M2) *Id* refers to sequence identity. SCG results are displayed as a ratio of supporting SCGs to a total number of detected SCGs. *pmoCAB* and *pxmABC* are operons coding for canonical and non-canonical particulate methane monooxygenase; *sMMO,* soluble methane monooxygenase; *mxaFI,* Ca^2+^-dependent methanol dehydrogenase, H_4_MTP, tetrahydromethanopterin pathway; H_4_F, methylene tetrahydrofolate pathway; RuMP, ribulose monophosphate pathway; and pSC, partial serine cycle.

## Data Availability

All the accession numbers of data generated in this study, such as raw reads and genome assemblies, are listed in Table 1. Good quality fragments of 16S rDNA sequences obtained by Sanger sequencing used to assign taxonomy were deposited in NCBI GenBank with the following accessions: PP327421 (*Methylobacter* sp. Wu1), OR939725 (*Methylomicrobium* sp. Wu6), and *Methylobacter* sp. Wu8 PP327423.
